# Supplementary material for: The leishmaniases in Kenya: A scoping review
Source: PLoS Negl Trop Dis. 2023 Jun 1;17(6):e0011358. doi: 10.1371/journal.pntd.0011358 (PMC10263336; doi:10.1371/journal.pntd.0011358)
Supplement: S1 Search Strategy — (PDF) [file pntd.0011358.s002.pdf]

PubMed:

("Leishmaniasis"[Mesh] OR "Leishmania"[Mesh] OR leishmaniasis OR leishmaniases OR leishmania OR kala-azar OR kala azar OR black fever[Text Word] OR oriental sore OR "Neglected Diseases"[Mesh] OR "Tropical Medicine"[Mesh] OR "Phlebotomus"[Mesh] OR phlebotomus)

AND

("Africa, Eastern"[Mesh] OR kenya OR kenyan OR kenyans OR "Global Health"[Mesh] OR global[Text Word] OR worldwide[Text Word])

EMBASE:

('leishmania'/exp OR leishmania OR 'leishmaniasis'/exp OR leishmaniasis OR leishmaniases OR 'kala azar' OR 'kala-azar' OR “black fever” OR “oriental sore” OR 'neglected tropical disease'/exp OR 'phlebotomus'/exp OR phlebotomus)

AND

('africa south of the sahara'/exp OR kenya OR kenyan OR kenyans OR 'global health'/exp OR global OR worldwide)

Web of Science:

TS=(leishman* OR kala azar OR "black fever" OR "oriental sore" OR phlebotomus OR neglected tropical disease*)

AND

TS=(“East Africa*” OR “Eastern Africa*” OR kenya* OR global OR worldwide)

Cochrane CENTRAL:

Search Number	Search	Results
#1	MeSH descriptor: [Leishmaniasis] explode all trees	467
#2	MeSH descriptor: [Leishmania] explode all trees	125
#3	MeSH descriptor: [Neglected Diseases] explode all trees	13
#4	MeSH descriptor: [Tropical Medicine] explode all trees	18
#5	MeSH descriptor: [Phlebotomus] explode all trees	7
#6	#1 OR #2 OR #3 OR #4 OR #5 OR leishmaniasis OR leishmaniases OR leishmania OR kala-azar or kala azar OR black fever OR oriental sore OR phlebotomus	1322
#7	MeSH descriptor: [Africa, Eastern] explode all trees	2661
#8	MeSH descriptor: [Global Health] explode all trees	165
#9	#7 OR #8 OR kenya OR kenyan OR kenyans OR global OR worldwide	73434
#10	#6 AND #9	239
