# Supplementary figures and images for: The leishmaniases in Kenya: A scoping review
Source: PLoS Negl Trop Dis. 2023 Jun 1;17(6):e0011358. doi: 10.1371/journal.pntd.0011358 (PMC10263336; doi:10.1371/journal.pntd.0011358)

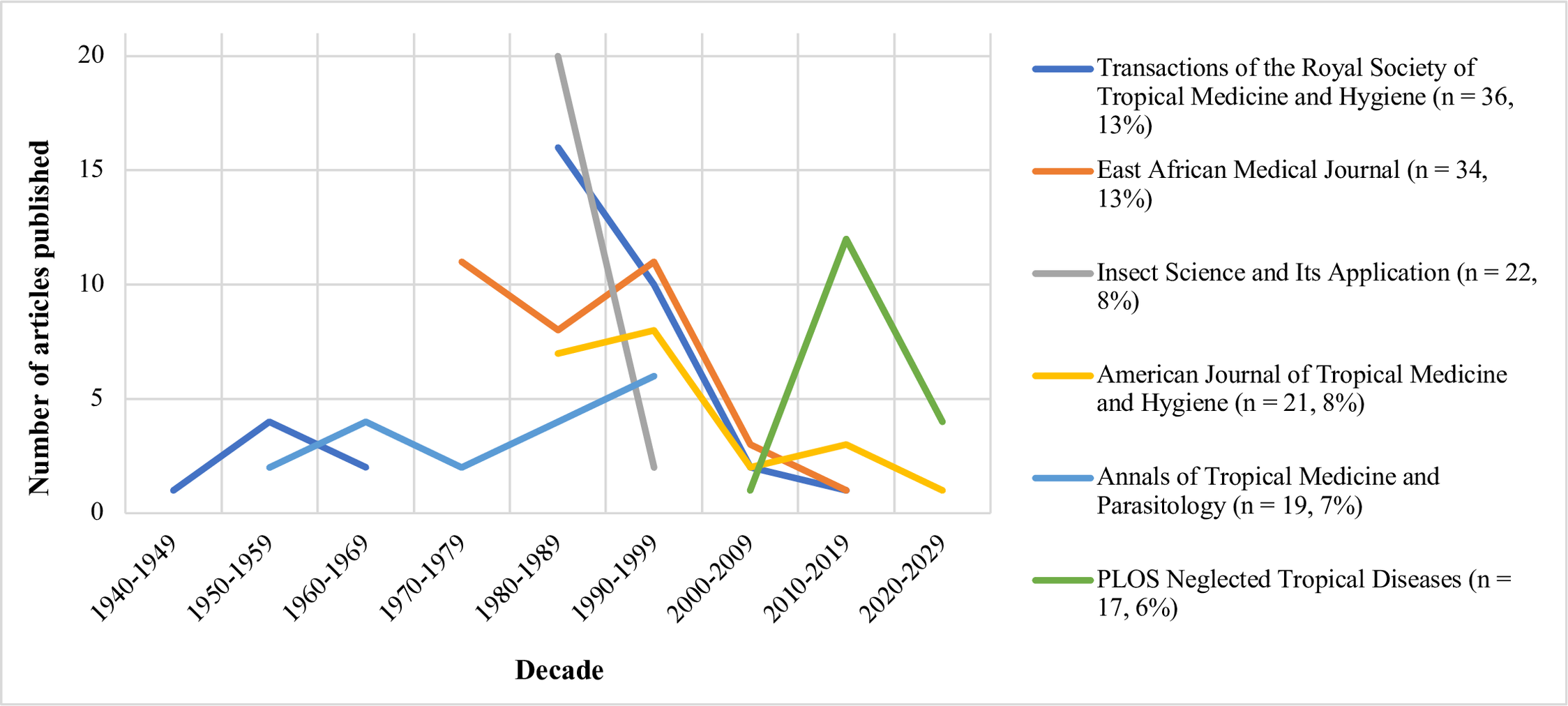

Supplement: S1 Chart — (TIF) [file pntd.0011358.s003.tif]

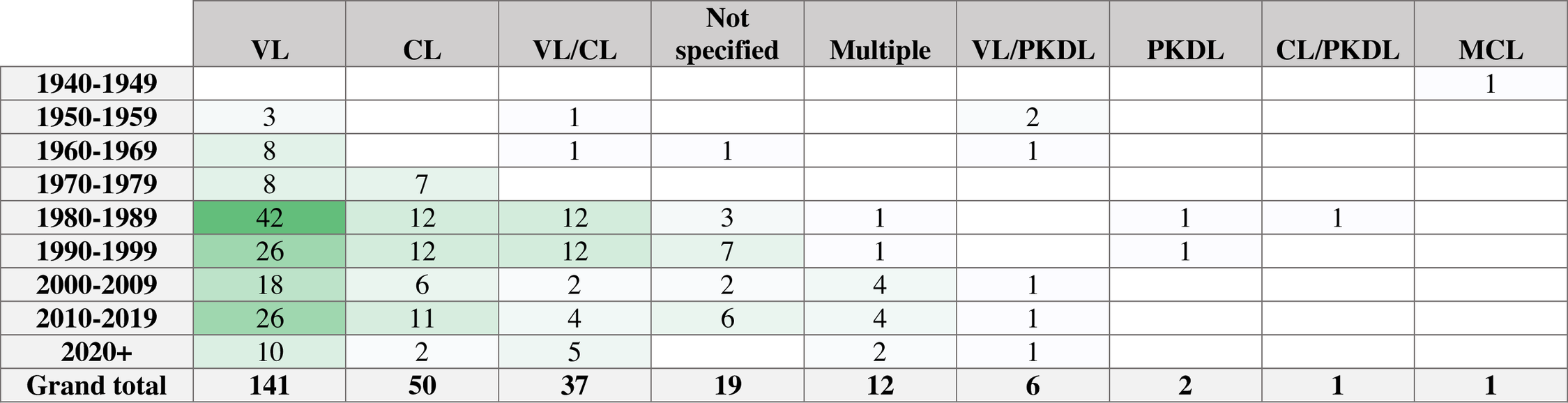

Supplement: S2 Table — (TIF) [file pntd.0011358.s006.tif]

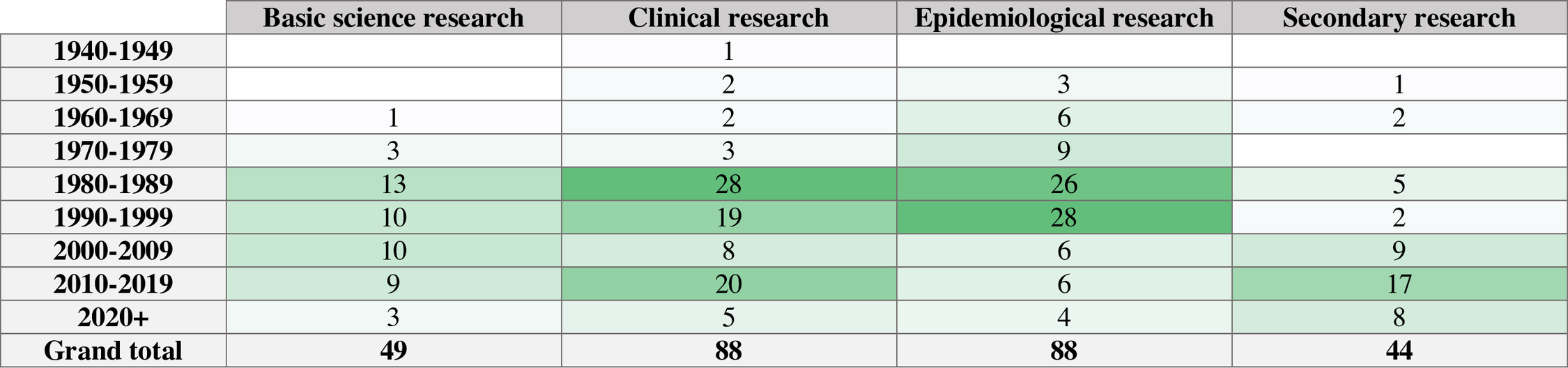

Supplement: S3 Table — (TIF) [file pntd.0011358.s007.tif]

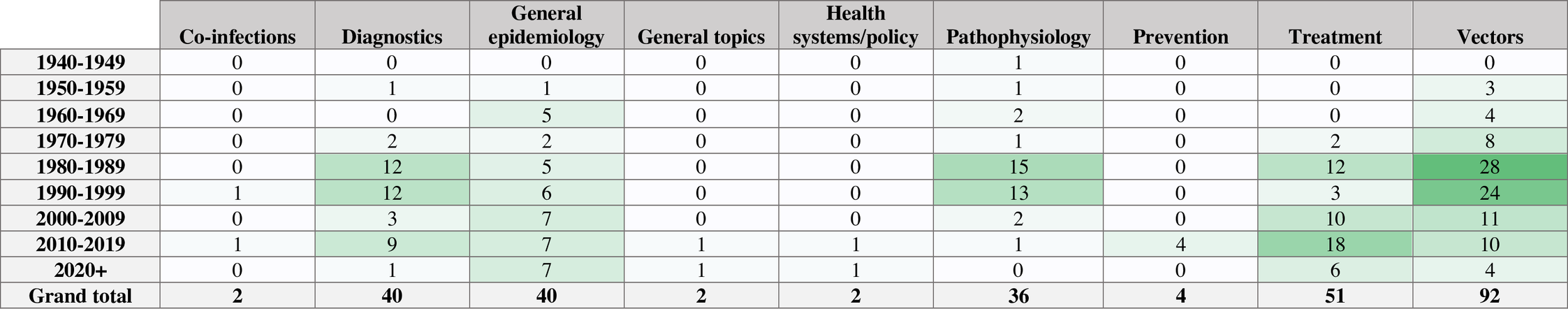

Supplement: S4 Table — (TIF) [file pntd.0011358.s008.tif]

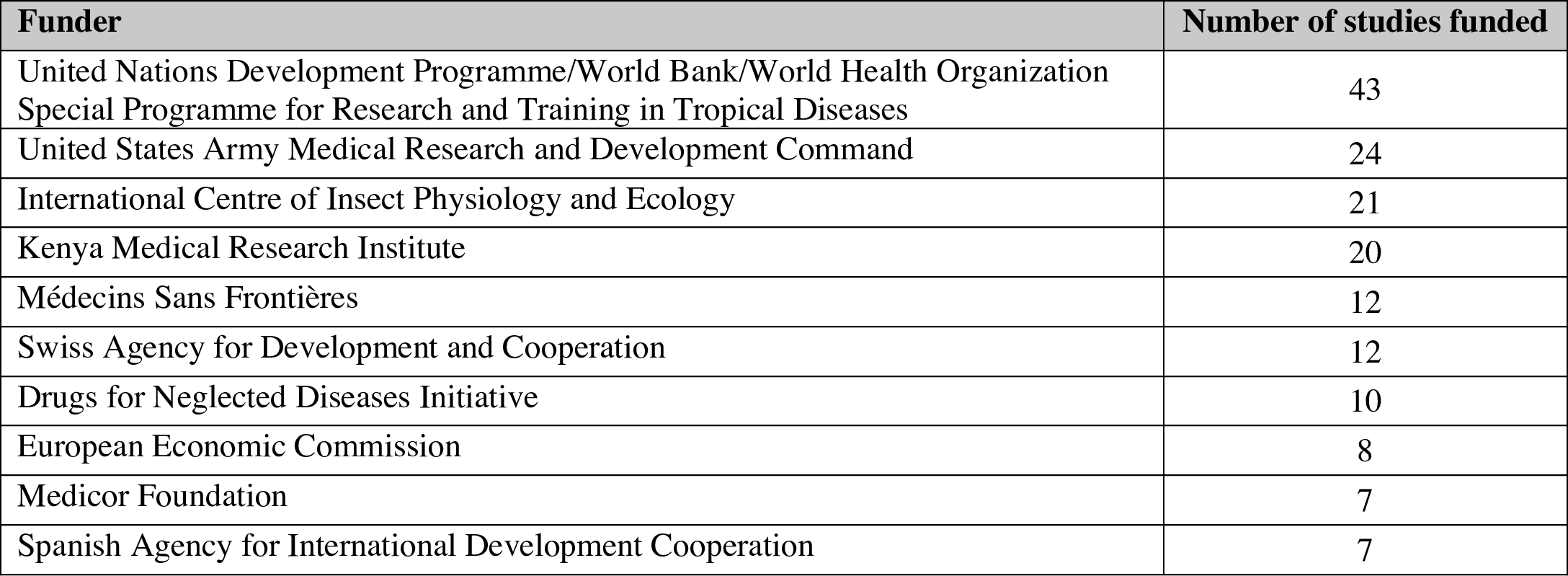

Supplement: S5 Table — (TIF) [file pntd.0011358.s009.tif]

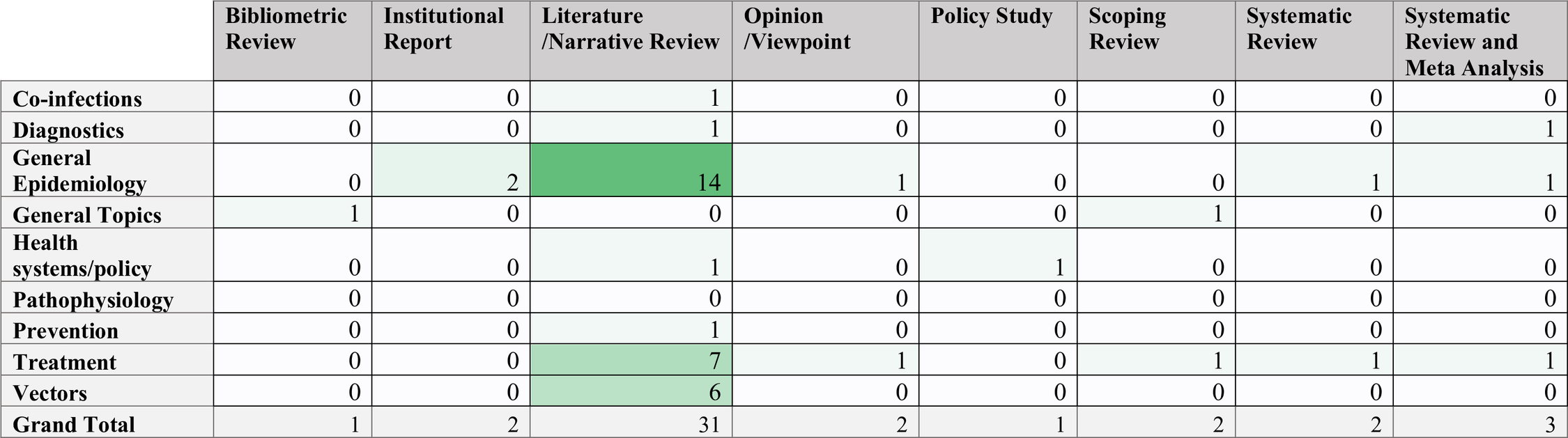

Supplement: S7 Table — (TIF) [file pntd.0011358.s011.tif]
