# Supplementary material for: The leishmaniases in Kenya: A scoping review
Source: PLoS Negl Trop Dis. 2023 Jun 1;17(6):e0011358. doi: 10.1371/journal.pntd.0011358 (PMC10263336; doi:10.1371/journal.pntd.0011358)
Supplement: S1 Table — (PDF) [file pntd.0011358.s005.pdf]

Type of data	Categories	Subcategory (if applicable)	Notes
**Type of leishmaniasis**	VL	-	If articles did not explicitly state a type of leishmaniasis, *Leishmania* species was used as a proxy given species are clinically associated with VL, CL, MCL and/or PKDL [1, 2, 3].
	CL	-	
	MCL	-	
	PKDL	-	
	VL/CL	-	
	VL/MCL	-	
	VL/PKDL	-	
	CL/MCL	-	
	CL/PKDL	-	
	MCL/PKDL	-	
	Multiple	-	This category was selected if 2+ types of leishmaniasis were discussed.
	Not specified	-	This category was selected if the type of leishmaniasis studied was not identified.
**Theme**	General epidemiology	-	Articles focused on the frequency, patterns, causes and/or risk factors of leishmaniasis in Kenya. If an article covered multiple themes from this table (ex. treatment, diagnostics, prevention, etc.) and one did not dominate over the others, then this theme was used.
	Prevention	-	Articles focused on preventing leishmaniasis. Examples include community education, behavior change, use of bed nets or repellents, environmental modifications (e.g., modifying house types or homesteads including animal enclosures), migration of humans or animals to areas without transmission, vaccines, preventative medications, etc.
	Pathophysiology	-	Articles focused on research related to human-pathogen interactions, clinical presentation of leishmaniasis in humans and/or manifestations of disease at sub-cellular/cellular/tissue/organ/organ system levels in humans.
	Diagnostics	-	Articles focused on diagnosis or monitoring of leishmaniasis using medical tests, clinical procedures, laboratory procedures, etc.
	Treatment	-	Articles focused on leishmaniasis treatment. Examples include medications, medical procedures, responses to therapy, clinical trials, etc.
	Health systems/policy	-	Articles focused on evaluating health systems and policies in Kenya. Examples include financing, training of health workers, procurement of supplies, etc.
	Vectors	-	Articles focused on anything related to the vectors that transmit leishmaniasis (parasites, sand flies, non-human animal reservoirs), unless the focus of the article was preventing vectors from transmitting leishmaniasis. If that was the case, the article was classified under the "prevention" theme.
	Co-infections	-	Articles focused on leishmaniasis co-infection with any other disease.
	General topics	-	Articles that do not fit into any other theme.
**Type of study**	Basic science research[b]	Animal study	Articles with research conducted within an animal model/system, where animals were raised/controlled in a laboratory setting.
		Cell study	Articles studying microorganisms, or human/ animal cells in vitro.

		Genomics/ Genetics/ Computational	Articles that conduct any analysis of genes or genomes related to leishmaniasis (note: must not focus on geographical distribution, that would qualify for "molecular epidemiology" subcategory under "epidemiological" category).
		Biochemistry	Articles that study protein, DNA, lipids, or carbohydrates using methods that do not better fit another category.
		Method development	Articles focused on the development of tools/techniques for further research/clinical application (note: if developing a diagnostic procedure, that qualifies for "diagnostic study" sub-category under "clinical" category related to diagnostic procedures).
		Other	Articles that do not fit under another sub-category.
	Clinical research[c]	Clinical study	Articles using interventional methods to assess health outcomes according to a research plan or protocol, including clinical trials. Interventions can be pharmacological, surgical or social.
		Diagnostic study	Articles that evaluate medical tests, markers, prediction models, decision tools and applications.
		Prognostic study	Articles that examine selected predictive variables or risk factors and assess their influence on the outcome of a disease.
		Case report/series/study	Articles that provide detailed report of the diagnosis, treatment and follow-up of an individual or group of patients, including chart reviews.
		Other	Articles that do not fit under another sub-category.
	Epidemiological research[d]	Molecular epidemiology	Articles that merge molecular biology into epidemiological studies for monitoring geographical distributions, reservoir status, etc.
		Method development	Articles that describe the development of epidemiological techniques.
		Cohort study	Articles that are prospective or retrospective studies that compare a particular outcome in groups of individuals.[a]
		Case-control	Articles that compare those with and without a disease.[a]
		Cross-sectional	Articles that analyze data collected at one point in time across a sample population.[a]
		Monitoring/ surveillance	Articles that aim to collect data about health, disease and their determinants in a population at a particular time or over a period of time, limited to counting the number of cases, animals, termite mounds, etc. using point-of-care techniques (e.g. rK39) or other tracking methods.
		Other	Articles that do not fit under another sub-category.
	Secondary research[e]	Meta-analysis	Articles that perform quantitative statistical analysis of studies to test the pooled data for statistical significance.[a]
		Systematic review	Articles that attempt to identify, appraise and synthesize all the empirical evidence that meets pre-specified eligibility criteria to answer a specific research question.[a]
		Scoping review	Articles that aims to map evidence on a topic and identify main concepts, theories, sources, and knowledge gaps.[a]
		Literature/narrative review	Articles that conduct comprehensive analysis of current knowledge on a topic without using a systematic methodology for identifying source literature.[a]
		Bibliometric review	Articles that summarize large quantities of bibliometric data to present the state of a research topic or field.[a]

		Policy studies	Articles that aim to understand how policies, regulations and practices may influence health.
		Opinion/viewpoint	Articles that present an accurate summary and critical analysis of an issue initiated by the authors and presents their opinions/view.
		Institutional report	Articles published by a health organization or governmental body.
**Year of publication**	-	-	The year the article was published as provided by article itself. Format: YYYY
**Funding Source**	-	-	Funding source was extracted if it was explicitly mentioned as some form of monetary support, including funding, financing, grants, etc. We did not interpret the terms "sponsor" or "supported" as meaning financial support. If no funding source was identified, we used "N/A". Format: full names, no acronyms
**Number of times cited**	-	-	We used the number provided by Google Scholar for each article. Format: ##

[a]Studies that self-identified as this type of study were categorized as such.
[b]The classification "Basic Science Research" was used if the article's main investigation took place in a laboratory.
[c]The classification "Clinical" was used if the article related to the observation or treatment of actual patients rather than theoretical or laboratory studies.
[d]The classification "Epidemiological" was used if the article related to the incidence, distribution, and control of leishmaniasis.
[e]The classification "secondary research" was used if the article analyzed data generated by other researchers or institutions.

References

1. The Strategic Plan for Control of Leishmaniasis 2021-2025. Kenya: Republic of Kenya Ministry of Health; 2021.
2. Cobo F. Imported Infectious Diseases: Woodhead Publishing; 2014.
3. Steverding D. The history of leishmaniasis. Parasit Vectors. 2017;10(1):82.
