# Supplementary material for: The leishmaniases in Kenya: A scoping review
Source: PLoS Negl Trop Dis. 2023 Jun 1;17(6):e0011358. doi: 10.1371/journal.pntd.0011358 (PMC10263336; doi:10.1371/journal.pntd.0011358)
Supplement: S6 Table — (PDF) [file pntd.0011358.s010.pdf]

Article	Number of times cited according to Google Scholar	Type of leishmaniasis	Theme	Type of study	Subcategory of study
Alvar J, Vélez ID, Bern C, Herrero M, Desjeux P, Cano J, et al.[1]	5111	VL/CL	general epidemiology	secondary research	literature/narrative review
Hotez PJ, Kamath A.[2]	1240	VL	vectors	secondary research	literature/narrative review
Guerin PJ, Olliaro P, Sundar S, Boelaert M, Croft SL, Desjeux P, et al.[3]	1057	VL	general epidemiology	secondary research	opinion/viewpoint
Dorlo TP, Balasegaram M, Beijnen JH, de Vries PJ.[4]	658	Multiple	treatment	secondary research	literature/narrative review
van Griensven J, Balasegaram M, Meheus F, Alvar J, Lynen L, Boelaert M.[5]	325	VL	treatment	secondary research	literature/narrative review
Feliciangeli MD.[6]	298	Multiple	vectors	secondary research	literature/narrative review
Harith AE, Kolk AH, Kager PA, Leeuwenburg J, Muigai R, Kiugu S, et al.[7]	292	VL	diagnostics	clinical research	diagnostic study
Chulay JD, Spencer HC, Mugambi M.[8]	211	VL/CL	treatment	clinical research	clinical study
Berman JD, Badaro R, Thakur CP, Wasunna KM, Behbehani K, Davidson R, et al.[9]	206	VL	treatment	clinical research	clinical study
Chunge CN, Owate J, Pamba HO, Donno L.[10]	202	VL	treatment	clinical research	clinical study

References:

1. Alvar J, Vélez ID, Bern C, Herrero M, Desjeux P, Cano J, et al. Leishmaniasis worldwide and global estimates of its incidence. PLoS One. 2012;7(5):e35671.
2. Hotez PJ, Kamath A. Neglected tropical diseases in sub-saharan Africa: review of their prevalence, distribution, and disease burden. PLoS Negl Trop Dis. 2009;3(8):e412
3. Guerin PJ, Olliaro P, Sundar S, Boelaert M, Croft SL, Desjeux P, et al. Visceral leishmaniasis: current status of control, diagnosis, and treatment, and a proposed research and development agenda. Lancet Infect Dis. 2002;2(8):494-501.
4. Dorlo TP, Balasegaram M, Beijnen JH, de Vries PJ. Miltefosine: a review of its pharmacology and therapeutic efficacy in the treatment of leishmaniasis. J Antimicrob Chemother. 2012;67(11):2576-97.
5. van Griensven J, Balasegaram M, Meheus F, Alvar J, Lynen L, Boelaert M. Combination therapy for visceral leishmaniasis. Lancet Infect Dis. 2010;10(3):184-94.
6. Feliciangeli MD. Natural breeding places of phlebotomine sandflies. Med Vet Entomol. 2004;18(1):71-80.
7. Harith AE, Kolk AH, Kager PA, Leeuwenburg J, Muigai R, Kiugu S, et al. A simple and economical direct agglutination test for serodiagnosis and sero-epidemiological studies of visceral leishmaniasis. Trans R Soc Trop Med Hyg. 1986;80(4):583-36.
8. Chulay JD, Spencer HC, Mugambi M. Electrocardiographic changes during treatment of leishmaniasis with pentavalent antimony (sodium stibogluconate). Am J Trop Med Hyg. 1985;34(4):702-9.
9. Berman JD, Badaro R, Thakur CP, Wasunna KM, Behbehani K, Davidson R, et al. Efficacy and safety of liposomal amphotericin B (AmBisome) for visceral leishmaniasis in endemic developing countries. Bull World Health Organ. 1998;76(1):25-32.
10. Chunge CN, Owate J, Pamba HO, Donno L. Treatment of visceral leishmaniasis in Kenya by aminosidine alone or combined with sodium stibogluconate. Trans R Soc Trop Med Hyg. 1990;84(2):221-5.
