# Supplementary material for: The leishmaniases in Kenya: A scoping review
Source: PLoS Negl Trop Dis. 2023 Jun 1;17(6):e0011358. doi: 10.1371/journal.pntd.0011358 (PMC10263336; doi:10.1371/journal.pntd.0011358)
Supplement: Attachment — Submitted filename: Response to Reviewers.pdf [file pntd.0011358.s012.pdf]

November 19, 2022

Dr. Richard Reithinger, Dr. Charles Jaffe
Academic and Section Editors
PLOS Neglected Tropical Diseases
Re: Decision on your PLOS Neglected Tropical Diseases submission

"The leishmaniases in Kenya: a scoping review"

By Grace Grifferty, Hugh Shirley, Katherine O'Brien, Jason L. Hirsch, Adrienne Orriols, Kiira Amechi, Joshua Lo, Neha Chanda, Sarra El Hamzaoui, Jorja Kahn, Samantha Yap, Kyleigh Watson, Christina Curran, Amina Atef Abdelalim, Neeloy Bose, Alissa Link Cilfone, Richard Wamai

Dear Dr. Reithinger and Dr. Jaffe,

Thank you for your consideration and preliminary review of our manuscript. We appreciate the feedback you have provided and the invitation to re-submit for full review. The team and I have revised the manuscript in accordance with your feedback. We have outlined in detail our responses to the review comments and included descriptions of the changes made below.

(1) REVIEWER'S EVALUATION: "The review is a review of the leishmaniases in Kenya. Among the 17 authors of the review, none has a Kenyan affiliation--please ensure that you have local researchers or policy makers represented, either by ensuring that dual affiliations are specified or that additional co-authors are invited."

AUTHORS' RESPONSE: We have added additional affiliations for several authors, which represent direct connections to Kenya and Eastern Africa (lines 12, 36, 37)

(2) REVIEWER'S EVALUATION: "Abstract/Results/Discussion: the findings that there is lack of research on MCL and PKDL should be tempered, given that prevailing Leishmania species in Kenya result in no MCL/PKDL, or extremely low prevalence of MCL/PKDL, i.e. that finding is, thus, not surprising."

AUTHORS' RESPONSE: We very much appreciate the reviewer for taking the time to review our manuscript and thank them for their comment. After discussion, the authors concur with the reviewer feedback. We have amended the manuscript in the abstract (lines 69-70, 83-84, 230-231 and 316-321).

(3) REVIEWER'S EVALUATION: "Please make sure to consult state-of-the-art reviews of the leishmaniases to ensure correct nomenclature, definitions, etc. (see: Reithinger R et al (2007) Cutaneous leishmaniasis. Lancet Infect Dis. 7: 581-596, or Burza et al. (2018) Leishmaniasis. Lancet 392: 951-970). Examples of inaccuracies are:
* Line 86: Phlebotomus and not Phlebotomine"

AUTHORS' RESPONSE:
The authors reviewed the Reithinger et al paper and appreciate the reviewer's expertise here. We determined that we would include this review as a reference to support several statements and corrected nomenclature as needed (lines 89 and 96).

(4) REVIEWER'S EVALUATION: "Line 87 - 90: there are pathologies as well, such as disseminated cutaneous leishmaniasis. Broadly speaking you have the cutaneous leishmaniases and visceral leishmaniasis. The former includes localized cutaneous leishmaniasis, disseminated cutaneous, and mucosal leishmaniasis. Visceral leishmaniasis includes visceral leishmaniasis and post-kala-azar dermal leishmaniasis, since PKDL is a manifestations seen in patients with visceral leishmaniasis after apparent clinical cure."

AUTHORS' RESPONSE: The authors concur with the inclusion of LCL and DCL in the intro (line 91). Additionally, the authors recognize the limitations of not using LCL and DCL classifications in a scoping review. Thus, the authors have added this as a limitation of the study in the limitations section (lines 386-389).

(5) REVIEWER'S EVALUATION: "Lines 91 onwards. You are mixing up species and sub-genera. Thus, it should be <MCL is frequently seen in L. Vianna spp.> (which includes Leishmania braziliensis)."

AUTHORS' RESPONSE: We agree and edited accordingly to improve clarity. Additionally, we removed L. braziliensis from examples of CL-causing parasites, as it is not seen in Kenya (line 96).

(6) REVIEWER'S EVALUATION: "Line 120: The sentence <Kenya, a burgeoning country in Sub-Saharan Africa, is among five East African countries that now bear the largest proportion of the global burden of leishmaniasis> cannot be correct, as there are a number of countries in LAC and Central Asia that have a much higher burden of cases) than Kenya /East Africa.
* Line 126: Please specify what you mean by <1,000 yearly>? Cases? Incidence per 1,000 population?"

AUTHORS' RESPONSE: The authors agree and have correct this line to read 'largest proportion of the global burden of VL,' as supported by the Alvar et al (2021) reference (line 125).

In response to the comment on line 126, the authors agree that there is an opportunity to better contextualize the burden of leishmaniasis by expanding and clarifying the existing point to include: "According to one estimate, there are approximately 1,600 cases of VL annually, resulting in the loss of over 13,000 DALYs and over 170 deaths. Recent estimates of leishmaniasis burden in Kenya place VL incidence over 1,000 yearly and combined CL and MCL incidence at nearly 600 yearly. The combined incidence of MCL and CL is estimated at over 580 annually, resulting in a loss of 253 DALYs ," (lines 129-131). Additionally, a phrase was added to the discussion to further clarify the distinction between reported cases and estimates (line 305).

(7) REVIEWER'S EVALUATION: "Please make sure that all figures are of adequate size and resolution. The full-page Figures 1- 4 are not very legible."

AUTHORS' RESPONSE: Larger image versions of Figures 1-3 have been re-uploaded to the submission portal. Figures 4A-D have been submitted as separate files to the manuscript submission portal, so each sub-figure may be viewed independently and more clearly. The font has also been increased in Figures 1-4. The authors have confirmed that the figures uploaded to the manuscript submission portal are within the recommended guidelines.

(8) REVIEWER'S EVALUATION: "Re gaps in knowledge / research--you may want to distinguish gaps that are more pertinent to the global level (e.g., basic biology, pathobiology, immunology, co-morbidities) versus those that are East Africa/Kenya specific (e.g., vector or reservoir ecology)."

AUTHORS' RESPONSE: The authors agree that specifying gaps in knowledge and research on a global vs East Africa/Kenya level is important and have amended the manuscript to reflect this. We have amended the manuscript accordingly by adding lines 336-346.

Yours sincerely,

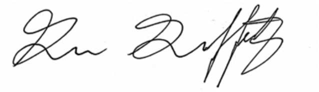

Grace Grifferty

University of Florida College of Medicine, Gainesville, FL, USA
Integrated Initiative for Global Health at Northeastern University, Boston, MA, USA
the African Center for Community Investment in Health, Nginyang, Baringo County, Kenya
