# Supplementary material for: The leishmaniases in Kenya: A scoping review
Source: PLoS Negl Trop Dis. 2023 Jun 1;17(6):e0011358. doi: 10.1371/journal.pntd.0011358 (PMC10263336; doi:10.1371/journal.pntd.0011358)
Supplement: Attachment — Submitted filename: Response to Reviewers_FINAL.pdf [file pntd.0011358.s014.pdf]

April 15, 2023

Dr. Richard Reithinger, Dr. Charles Jaffe
Academic and Section Editors
PLOS Neglected Tropical Diseases
Re: Decision on your PLOS Neglected Tropical Diseases submission

"The leishmaniases in Kenya: a scoping review"

By Grace Grifferty, Hugh Shirley, Katherine O'Brien, Jason L. Hirsch, Adrienne Orriols, Kiira Amechi, Joshua Lo, Neha Chanda, Sarra El Hamzaoui, Jorja Kahn, Samantha Yap, Kyleigh Watson, Christina Curran, Amina Atef Abdelalim, Neeloy Bose, Alissa Link Cilfone, Richard Wamai

Dear Dr. Reithinger and Dr. Jaffe,

We thank you for the opportunity to re-submit our manuscript to your esteemed journal. We appreciate your time and effort in reviewing our manuscript and providing valuable feedback. The authors found the comments and suggestions from the reviewers to be immensely helpful in improving the quality of the manuscript and believe that the revised version addresses the reviewers concerns and suggestions. We hope that you will find the revised manuscript to be of satisfactory quality for publication in your journal. Below you will find our detailed responses to all the reviewers' comments.

Reviewer #1:

1) "Leishmaniasis is a topic that has been widely researched in Kenya, considering the volume of the publications that have me the eligibility criteria of this scoping review. The conclusions of this paper would be important to shape policy in Kenya, and as such the authors should have considered including the input of local policy makers in this kind of paper, especially in helping explain the apparent paucity of data on some aspects of Leishmaniasis research. This will give this paper a stronger contextual basis and strengthen the basis of the conclusions. The authors should be seen to have collaborated with local based policy makers and researchers for context."

AUTHORS' RESPONSE: We concur having Ministry of Health (MOH) representation in the authorship could add value. The senior author (RW) has been working closely with Kenya's MOH and other key stakeholders (i.e., icipe, Kemri, DND*i*) on the leishmaniases for the past 10 years. He has been in the national Technical Working Group that developed the Strategic Plan for Control of Leishmaniasis 2021-2025, and the 2017 National Guidelines for Health Workers for Diagnosis, Treatment and Prevention of Visceral Leishmaniasis; the TWG is the lead group advising the MOH on national programming. Part of his VL and CL work in Kenya is funded by the Kenya government in partnership with icipe and Kemri. When the UK-FCDO ASCEND program, which included support for VL in East Africa, was discontinued early in 2021, and The END Fund bridged the gap commencing for the first time to work on VL in East Africa, RW was requested to step in as technical advisor and conducted a baseline review of the VL programs in the region; the unpublished review has been disseminated in regional meetings. The African Centre for Community Investment in Health (ACCIH) that has been running a VL treatment center since 2019 in VL endemic Baringo County works closely to advise the regional MOH. Finally, Kenya national VL focal person at MOH, Dr. Davis Wachira, with whom RW and the ACCIH team worked closely for nearly 10 years and would have been the appropriate MOH contributor given the longevity and history of work for this manuscript has since passed away (May 2022). Given Wachira's contribution to the national leishmaniasis program for many years and to RW's relationship with the MOH, we would be honored to include him in the authorship if this is acceptable by the journal We recognize such an authorship may be feasible as Wachira fulfils only the first authorship criteria of the International Committee of Medical Journal Editors (ICMJE) and as expounded in "The Authorship of Deceased Scientists and Their Posthumous Responsibilities." This notwithstanding,

we have concurred it is appropriate to add Wachira's name in the acknowledgements (lines 440-443).

2) "It would be useful if the authors would update the search strategy to include papers published in the last one year (since January 2022), given that this is a widely researched topic in Kenya. In the last few years, many publications have explored the impact of COVID-19, for example, on most neglected tropical diseases. A lot of these papers would have been published in the recent few years. It would be key to state reasons why this scoping review was not updated to the most recent data.

AUTHORS' RESPONSE: We thank the reviewer for this feedback. On March 16, 2023 the authors conducted a comprehensive query using the same databases and parameters as in the manuscript. We determined that if articles published in February 2022 and later were included, then 950 additional articles would need to be screened. This is a large undertaking that would require significant overhaul of the manuscript and delay. As such, the authors believe that it would be best to leave these articles as an opportunity for future publications to explore. Furthermore, in the time between undertaking evaluation of these recent studies and subsequent resubmission, there would inevitably be other studies published potentially validating another update, if this logic was followed. The time lag between our cut-off search in January 2022, screening and analysis to submission in September 2022 is appropriate.

Importantly, we see that the primary rationale of the reviewer is the potential omission of studies exploring impact of the Covid-19 pandemic on VL programming in Kenya. We would like to assure the reviewer that no such Kenya-specific studies exist. The senior author (RW) is involved in VL work at multiple levels in the region as an expert advisor to implementing groups including the Ministry of Health, is a member of VL research groups, and as lead of ACCIH that has been running a VL treatment center since 2019. RW has been involved in several global, regional (East Africa) and national (Kenya) activities related to VL and CL and is well-aware of the research context. Studies that would have been published during the Covid-19 pandemic would have been available for presentation, discussion and policy guidance during key events in the last 3 years in which RW participated or was represented. These include the World7Leish congress in Cartagena Colombia (August 2022), the END Fund's 10th Anniversary Learning Summit in Nairobi, Kenya (May-June 2022), World Health Organization's (WHO) Programme Review Meeting of South Asia and East Africa: progress, challenges and next steps (June, 2022), and WHO's Stakeholders' *meeting for the Development of a Strategic Plan for the Elimination of Visceral Leishmaniasis in East Africa*, in Nairobi, Kenya (24 - 27 January 2023).

This notwithstanding, our cut-off in January 2022 allowed inclusion of articles that would have been published during 2 years of the Covid-19 pandemic at that time. In lines 371-372 we did acknowledge the effect of Covid-19 citing one study, thus "Lastly, the COVID-19 pandemic likely inhibited research and operations, so we expect a decrease in publications from 2020-2022". VL programming in Kenya and rest of East Africa (including in our own work with ACCIH) was impacted by the pandemic, but also by the early discontinuation of the UK-FCDO ASCEND program in 2021. Following the ending of ASCEND, The END Fund has bridged the gap on VL in East Africa. As Uwishema et al., point out, the Covid-19 pandemic was expected to have an impact in programming. However, no specific data is presented for any of the countries in their study [Uwishema O, Sapkota S, Wellington J, Onyeaka CVP, Onyeaka H. Leishmaniasis control in the light of the COVID-19 pandemic in Africa. *Ann Med Surg (Lond)*. 2022;80:104263. doi:10.1016/j.amsu.2022.104263]

3) "The authors should explicitly explain the basis of categorizing the articles included in the review in the various 'themes' and subgroups, and why it is important for this paper. Ordinarily, some fields of research would attract fewer papers (e.g. reviews, since one article usually summarizes many other publications). Therefore, the metrics of comparison using proportions would be grossly misleading, since some of the fields would naturally be underrepresented."

AUTHORS' RESPONSE: The authors agree that it is important to explicitly explain the basis of categorizing the articles included in a review and the rationale behind the chosen themes and subgroups. As a result, we have added the following sentence to lines 204-206: "Themes and subthemes were developed to facilitate categorization of papers into a framework that would allow quantitative comparisons between study design and focus over time."

Regarding the potential bias in comparing the proportions of articles across different types of studies, particularly secondary research, the authors acknowledge that some fields may naturally attract fewer articles. However, this does not necessarily invalidate the use of proportions as a metric of comparison. Rather, it highlights the need for careful consideration of the context and the goals of the secondary research. Taking both this and the reviewer's comments into consideration, the authors have added an additional figure in the appendix (S11 Figure) that dives deeper into the nature of the secondary research articles.

In addition, the authors have added the following to line 263-270: "It is important to consider, however, that some types of studies naturally attract fewer papers, most notably those in the secondary research category including meta-analyses, systematic reviews, scoping reviews, literature/narrative reviews, bibliometric reviews, and others. This may be one reason why the secondary research category contains the fewest number of articles. To further evaluate the nature of the secondary research, S11 Figure was created. S11 reveals that the majority of literature/narrative reviews covered the theme general epidemiology (n = 14, 45%), followed by treatment (n = 7, 22%) and vectors (n = 6, 19%). Lastly, there was no secondary research found focusing solely on prevention."

4) "In order to avoid a classification bias and to identify additional areas that have not been researched, the authors ought to have identified and contextualized (from outset) what areas of Leishmaniasis research are topical/relevant given the stage of control/elimination in Kenya. In this way, the authors would ensure that areas that are totally missing from publications are highlighted. For example, studies on modeling the burden/distribution, economic costs and risk mapping of Leishmaniasis in Kenya would be important for the elimination efforts in Kenya. This scoping review is silent ai any of these types of studies."

AUTHORS' RESPONSE: The authors appreciate the reviewer's feedback and concur that there is a degree of classification bias in this study. To limit classification bias from the onset, the authors determined the "themes" to be used prior to beginning the screen. After the full-text screen was complete, a pilot study was conducted to create a codebook (S3 Table) and further classify included articles by type of leishmaniasis, type of study, and subcategory of study. The authors felt this was necessary to provide a more comprehensive analysis of the collected articles. However, it was at this stage that classification bias was present as these classifications were determined after having read through the full texts in the prior stage. To account for this, the following text has been added to lines 415-422 of the limitations section:

"The authors acknowledge that there was a degree of classification bias in this study. To limit classification bias from the onset, the authors determined the "themes" to be used prior to beginning the screen. After the full-text screen was complete, a pilot study was conducted to create a codebook (S3 Table) and further classify included articles by type of leishmaniasis, type of study, and subcategory of study. The authors felt this was necessary to provide a more comprehensive analysis of the collected articles. However, it was at this stage that classification bias was present as these classifications were determined after having read through the full texts in the prior stage. However, the classification we used enabled us to identify topics of research relevant to the policy on the elimination of VL in Kenya."

Regarding the reviewer's comments about "modeling the burden/distribution, economic costs and risk mapping", the authors assure the reviewer that these were represented in these categories as

described in S3_table: monitoring/surveillance and molecular epidemiology (subcategories of type of study) and health systems/policy (theme). Indeed, such studies provide important relevance for elimination policies. Regarding elimination policies, the authors have expanded upon this topic further in the author's response to reviewer comment #21 in this letter.

5) "The authors should consider proofing the document for grammar and spelling prior to resubmission."

AUTHORS' RESPONSE: We very much appreciate the reviewer for taking the time to review our manuscript in such detail. As the reviewer suggested, several authors have now re-read the manuscript and corrected grammatical and spelling errors.

6) "Line 52- Reference is made to 'types' of leishmaniasis. I would rather call these forms of presentation of the disease as opposed to types."

AUTHORS' RESPONSE: The authors agree that it would be helpful to clarify that "types of leishmaniasis" we refer to are the various forms of clinical presentation of leishmaniasis. We have added this clarification to lines 219/220.

7) "The extent of leishmaniasis research is undescribed'. What does this mean? From the number of articles pulled out in the scoping review, it would seem this is a topic that has been widely researched in Kenya (>250 articles meeting criteria)."

AUTHORS' RESPONSE: In this sentence, we are referring to the fact that no other scoping reviews have been conducted that focus solely on Leishmaniasis in Kenya. We have revised this statement in lines 54/55 to now read "the extent of leishmaniasis research has not yet been systematically described."

8) "Line 58- Please write 'PRISMA' in full first time it is used"

AUTHORS' RESPONSE: Thank you for your feedback. We have spelled out "PRISMA" in lines 59 and 156.

9) "The scoping review covers until January 2022 which is more than 1 year ago. Would the authors want to update this search to be more current. This is especially so because it seems this is a widely researched topic."

AUTHORS' RESPONSE: The authors have previously responded to this comment in authors' response #2.

10) "Conclusions line 69- Could it be that the minimal research on MCL and PKDL in Kenya is because these forms are rare? In fact, this has been acknowledged by the authors later in the manuscript (line 136/316). Please explain"

AUTHORS' RESPONSE: The authors fully agree with the reviewer's comment. Due to the word limit of the abstract, we are unable to elaborate further on this point within the abstract but have addressed the reviewer's concern in lines 352-354 of the manuscript: "PKDL incidence in Kenya is unknown although reported as suspected by some treatment centers. For this reason, it is recognized in the national strategic plan as of importance in controlling VL, and as such we recommend PKDL incidence studies be conducted [22]."

11) "Line 106- Non-specific presentation is mainly common in the early stages of the disease. The later stages are almost clinically pathognomonic e.g. the disfiguring skin lesions of CL/MCL and the organ

enlargement of VL. The authors should consider making this clear. In the middle- and low-income countries, lack of diagnostics is a major hindrance to early treatment."

AUTHORS' RESPONSE: The authors agree with this clarification and added "early in the course of each disease" to line 107.

12) Line 117- It would be important for the authors to indicate that most of the drug treatments are expensive and unavailable in places like Kenya, where hospitals rely on donations from WHO for these medications. This is important for context.

AUTHORS' RESPONSE: We agree with the reviewer's suggestion that this provides important context and so have added "Nevertheless, treatment for the leishmaniases can be prohibitively expensive, and providers often rely on the donation of supplies and medications to care for their patients." to lines 134-135.

13) Line 134- The authors should consider citing some of the primary literature on CL in Kenya e.g. https://journals.plos.org/plosntds/article?id=10.1371/journal.pntd.0007712, https://pubmed.ncbi.nlm.nih.gov/31971945/,

AUTHORS' RESPONSE: We agree that citing primary literature on CL prevalence in Kenya may be helpful. We cite the 2021 Strategic Plan for Control of Leishmaniasis by the Kenyan Ministry of Health, which has a more comprehensive review of the active disease foci for CL, which we believe provided the necessary context for the statement made in lines 153-154. Having reviewed the suggested article, we find it is a useful contribution to the literature and have included it by adding the line "with a recent outbreak investigation in Nakuru county identifying a potentially large burden" and citing the appropriate article in lines 147-148.

14) "Line 157- Please proofing the sentence for correct grammar. The word 'and' could have been missed."

AUTHORS' RESPONSE: After reviewing the sentence, the authors maintain that this sentence is written correctly. The phrase "a tool to facilitate independent article screening" is describing the tool Rayyan, which is mentioned directly before. Therefore, the word "and" is not needed before the word "results".

15) "Article inclusion and exclusion criteria- The authors should specify if dissertations were included among the eligible articles. If not, the authors should justify"

AUTHORS' RESPONSE: Thank you for this observation. If a dissertation was published in a peer-review journal with the traditional article structure (introduction, methods section, results, conclusion/discussion) then it was considered for inclusion if it also met the other criteria described in Fig 3. We have added the text "Grey literature was excluded." to line 197 to further clarify this point.

16) "Line 191- Please explain what is meant by "Themes". How were these established? Please provide more information on the basis of categorization of the articles by themes."

AUTHORS' RESPONSE: The authors appreciate the reviewer's question and thank them for the opportunity to respond. We decided to use the word "theme" because it is defined as the central topic of a piece, and our goal was to classify each article by identifying its main topic. In the absence of a pre-established method for categorizing leishmaniasis research, we developed our own. These "themes" were chosen after lengthy discussion between the authors to determine the main thematic areas of Leishmaniasis research, and chose the terms based on what would allow us to account for a wide range of research topics. Please see S3 Appendix for a detailed description of each theme.

17) "The authors should explicitly explain the basis of categorizing the articles in the various themes and sub groups, and why it is important for this paper. Ordinarily, some fields of research would attract fewer papers (e.g. reviews, since one article usually summarizes many other publications). Therefore the metrics of comparison using proportions would be misleading, since some of the fields would naturally be underrepresented."

> **AUTHORS' RESPONSE:** The authors have previously addressed this comment in the authors' response #3 and #16 above.

18) "The authors should explain if they also considered studies on modeling the burden/distribution, economic costs and risk mapping of Leishmaniasis in Kenya. Such studies would be important for the elimination efforts in Kenya. This scoping review is silent on any of these types of studies. This major limitation should be acknowledged by the authors."

> **AUTHORS' RESPONSE:** The authors have previously addressed this comment in the authors' response #4 above.

19) "This paper highlights the areas in Leishmaniasis research that are under-studied. It would be even better if the actual gaps in the research were highlighted. For example, it would be important to know what actual gaps are highlighted under diagnostics, treatment, prevention etc, as these will be more useful for the Leishmaniasis control effort in Kenya. The authors should update to include this information or explain why this data is not included."

> **AUTHORS' RESPONSE:** The authors agree with the reviewer that identifying the specific gaps within the themes would be beneficial. However, doing so would require in-depth analysis of each theme and each article within each theme. This is because identifying each gap would require a unique set of research questions, methodologies and results to effectively understand the issue. For example, identifying gaps in leishmaniasis diagnostics would require an in-depth analysis of the current diagnostic methods and their limitations, followed by a discussion of the specific diagnostic needs for different populations and clinical forms of the disease. It is our view that such an analysis would be complex and warrants its own manuscript to fully explore and address the gap.

20) "Line 303- data from DHIS2 is aggregated, and is possible that same patient with CL lesions/VL symptoms could be reported each time they visit the hospital for follow up. Therefore, this dataset would not be accurate for highlighting the 'apparent increased burden' of Leishmaniasis"

> **AUTHORS' RESPONSE:** We appreciate this comment. The knowledge and experience of one of the authors (RW) who was involved in development of DHIS2 in Kenya and uses it (at the ACCIH) is that this tool captures unique patient records discounting duplicate cases. At VL treatment centers in Kenya, Health Records Information Officers (HRIOs) who were trained in DHIS2 are the ones who summarize VL from facility records and then input it into the DHIS2. Treatment for VL and CL takes many days (for most cases, about 2 weeks to a month for VL and up to 6 months for CL). DHIS2 captures treatment outcomes such as relapse or treatment failure separately. Furthermore, in Kenya there is limited co-occurrence of CL and VL in the same area. Thus, we expect no such overlapping duplicates of reports for Kenya. In addition, in this paragraph we note recent reported outbreaks [see also, Ngere I, Gufu Boru W, Isack A, et al. Burden and risk factors of cutaneous leishmaniasis in a peri-urban settlement in Kenya, 2016. *PLoS One*. 2020;15(1):e0227697. Published 2020 Jan 23. doi:10.1371/journal.pone.0227697; Mewara A, Gudisa R, Padhi BK, Kumar P, Sah R, Rodriguez-Morales AJ. Visceral leishmaniasis outbreak in Kenya-a setback to the elimination efforts. *New Microbes New Infect*. 2022;49-50:101060. Published 2022 Nov 29. doi:10.1016/j.nmni.2022.101060]. Finally, one of the authors (RW) is well aware of the data trends and issues in Kenya given the nature of his work (see response to #1 and 2).

21) "The authors should summarize the implications of their findings to policy and prevention efforts in Kenya"

AUTHORS' RESPONSE: The authors concur that it would be beneficial to further elaborate on the implications of our findings to policy and prevention efforts in Kenya As a result, we have added the following text to lines 323-332: "In January 2023, the WHO's Stakeholders' meeting for the Development of a Strategic Plan for the Elimination of Visceral Leishmaniasis in East Africa (attended by RW and KO) determined that VL in East Africa is eliminable by 2030, which is in line with the NTDs 2030 roadmap. Key discussions focused on key needs in research including reviews precisely like what we have done. Kenya will be the first country to have conducted a systematic review of existing research on leishmaniases. Technical experts recognize the value for such studies to inform research prioritization for elimination targets. The call for operational and implementation research for optimizing existing interventions in diagnosis, treatment, health systems and vector control can be validated for Kenya by the findings of our review [20]. This review will not only fill a major gap in Kenya, but also serve as a model for similar reviews in other countries in the region given the common strategy now under development."

22) "Consider removing the % labels on the data in fig 4A and 4B"

AUTHORS' RESPONSE: After discussion, the authors feel that it is important to keep the % labels as it provides context for each data point in relation to the larger dataset. We think this allows easier reference and interpretation of the chart.

23) "S3 table- please perform a grammar check on the table cells to remove errors. For example, the word 'given' is misspelt as 'gjven'"

AUTHORS' RESPONSE: The authors have corrected the grammatical and spelling errors found in the table.

<u>Reviewer #2:</u>

1) Line 96: the specific mentioning of L. tropica for CL is quite arbitrary, as L. major and L. braziliensis are also among the most prevalence species.

AUTHORS' RESPONSE: To reflect that our paper is focused on parasites relevant to Kenya, we made several edits to clarify this line. We changed "VL is more frequently seen with certain species, such as *L.donovani*, while CL is seen with *L. tropica,* and MCL is frequently seen in *L. Viannia subgenus"* to "VL in Kenya is more frequently caused by certain species, such as *L.donovani*, while CL is seen with *L. tropica* [3,5]." This is not meant to be a comprehensive list of parasites and their associated clinical presentations, which can be found at the papers cited in lines 96-97.

2) Line 96: MCL is also seen in L. aethiopica (which has potential relevance for Kenya).

AUTHORS' RESPONSE: See above.

3) Line 111: Skin scrapes and fine needle aspiration are more frequently used methods for harvesting Leishmania than punch biopsies.

AUTHORS' RESPONSE: We appreciate this feedback and have added "skin scrapings, fine-needle aspirations, or punch biopsies of the ulcerated skin or mucosal lesions, allowing…" to reflect this.

4) Line 118: Pentamidine is not recommended for treatment of VL, due to its toxicity in therapeutic regimens. In East Africa paromomycin is used as first line treatment in combination with SSG, and miltefosine is also used as second line (in combination with liposomal amphotericin.

AUTHORS' RESPONSE: We edited lines 125-127 in response to this expert comment and in with review of the literature to: "Treatment regimens for VL in East Africa include pentavalent antimonials such as sodium stibogluconate plus paromomycin for 17 days. Second line therapies include formulations of amphotericin B or miltefosine."

5) Line 120: topical paromomycin is not commercially available. Pentavalent antimonials are the mainstay in Old World CL, and since last year PAHO has recommended miltefosine as first line treatment of New World CL.

AUTHORS' RESPONSE: Thank you for this comment. It is true that the topical paromomycin, is not commercially available, but it was included in the 2010 WHO guidelines (citation #12) for the treatment or CL. As the review focuses on Kenyan leishmaniases, the text reflects recommended treatment regiments for Old World CL from the WHO's most recent guidance. In addition, we have added text to lines 125-127 regarding this topic.

6) Line 305: GBD needs to be spelled out (Global Burden if Disease?)

AUTHORS' RESPONSE: The authors have spelled out GBD in line 336.

7) Line 319: authors mention a prevalence (should this not be incidence?) of 2-5%. However, this is just an estimation. A recent clinical trial in East Africa (Musa et al, Clin.Inf.Dis. 2022) found no PKDL in Kenya in a 6 months follow up after treatment of VL. Because the PKDL incidence is not known, and because of the public health implications of PKDL, I would recommend authors to make a recommendation for PKDL incidence studies.

AUTHORS' RESPONSE: The authors confirmed that the articles we referenced pertaining to the 2-5% data point refer to that data as "prevalence," and thus we maintain the use in this instance. However, we concur with the reviewer and have added this text to lines 352-354: "PKDL incidence in Kenya is unknown although reported as suspected by some treatment centers. For this reason, it is recognized in the national strategic plan as of importance in controlling VL, and as such we recommend PKDL incidence studies be conducted."

8) Line 324: Rightfully HIV is mentioned as an important co-infection. However, TB is another important co-infection, due to the relatively high prevalence of pulmonary and extra-pulmonary TB in VL patients (re-activation of latent TB infection due to VL-caused immune-suppression), and the fact that undiagnosed TB is a main reason for VL treatment failure.

AUTHORS' RESPONSE: The authors agree that TB-VL co-infection is an important topic that should be discussed in our manuscript. We have added relevant text to lines 109 and 359.

Lines 109-115: "Tuberculosis (TB) co-infection can complicate treatment further. Studies have estimated that the prevalence of TB in HIV-VL co-infected individuals may be between 5.7% and 29.7% [11]. TB infection leaves the host more susceptible to Leishmania infection, creating a co-infection (often TB-VL) that can lead to poor prognosis [12,13]. Because the Leishmania parasite and the tuberculosis bacterium employ similar models of macrophage inhabitation, and thus trigger similar immune responses from the host, co-infection is challenging to diagnose and often remains undiscovered for years [13]."

Line 360: "VL-TB co-infection is also chronically under researched and goes un-mentioned in the 2021-2025 Kenya Strategic Plan for Control of Leishmaniasis [22]. There are various case studies published on VL-TB and CL-TB co-infection outside of East Africa, showing the complexities and success of treatment [33-35]. More robust, quality data is needed in East Africa given that TB presents a barrier to successful VL treatment."

9) The tables and figures are clear, but I hope the final print of figure 4 will have a better readable font.

AUTHORS' RESPONSE: Figure 4A-D has been submitted in the portal as four separate high quality figures, which should ameliorate the font issue. The figure in the manuscript draft is a lower-quality screenshot of all four figures in one.

The authors declare no conflict of interest. All authors have reviewed and approved the final manuscript and confirm that the manuscript is not under consideration by another journal. We thank you for your evaluation of our manuscript.

Yours sincerely,

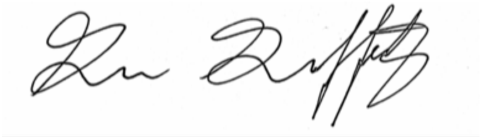

Grace Grifferty

University of Florida College of Medicine, Gainesville, FL, USA
Integrated Initiative for Global Health at Northeastern University, Boston, MA, USA
the African Center for Community Investment in Health, Nginyang, Baringo County, Kenya
