# Supplementary material for: The leishmaniases in Kenya: A scoping review
Source: PLoS Negl Trop Dis. 2023 Jun 1;17(6):e0011358. doi: 10.1371/journal.pntd.0011358 (PMC10263336; doi:10.1371/journal.pntd.0011358)
Supplement: Attachment — Submitted filename: Leismaniases in kenya; Scoping review.docx [file pntd.0011358.s013.docx]

**Title: The leishmaniases in Kenya: a scoping review**

*Summary and overall comments*

This is a scoping review covering Leishmaniases in Kenya, an important public health problem that is often neglected. The authors have rigorously reviewed publications between the 1980s and January 2022. The findings of this review, I believe, would be impactful for policy makers and Leishmaniasis prevention efforts in Kenya. The authors should consider the following suggestions to improve the quality of this publication;

1. Leishmaniasis is a topic that has been widely researched in Kenya, considering the volume of the publications that have me the eligibility criteria of this scoping review. The conclusions of this paper would be important to shape policy in Kenya, and as such the authors should have considered including the input of local policy makers in this kind of paper, especially in helping explain the apparent paucity of data on some aspects of Leishmaniasis research. This will give this paper a stronger contextual basis and strengthen the basis of the conclusions. The authors should be seen to have collaborated with local based policy makers and researchers for context.

2. It would be useful if the authors would update the search strategy to include papers published in the last one year (since January 2022), given that this is a widely researched topic in Kenya. In the last few years, many publications have explored the impact of COVID-19, for example, on most neglected tropical diseases. A lot of these papers would have been published in the recent few years. It would be key to state reasons why this scoping review was not updated to the most recent data

3. The authors should explicitly explain the basis of categorizing the articles included in the review in the various ‘themes’ and subgroups, and why it is important for this paper. Ordinarily, some fields of research would attract fewer papers (e.g. reviews, since one article usually summarizes many other publications). Therefore, the metrics of comparison using proportions would be grossly misleading, since some of the fields would naturally be underrepresented.

4. In order to avoid a classification bias and to identify additional areas that have not been researched, the authors ought to have identified and contextualized (from outset) what areas of Leishmaniasis research are topical/relevant given the stage of control/elimination in Kenya. In this way, the authors would ensure that areas that are totally missing from publications are highlighted. For example, studies on modeling the burden/distribution, economic costs and risk mapping of Leishmaniasis in Kenya would be important for the elimination efforts in Kenya. This scoping review is silent ai any of these types of studies.

5. The authors should consider proofing the document for grammar and spelling prior to resubmission.

*Specific Comments*:

**Abstract**

Line 52- Reference is made to ‘types’ of leishmaniasis. I would rather call these forms of presentation of the disease as opposed to types

Line 54- ‘The extent of leishmaniasis research is **undescribed**’. What does this mean? From the number of articles pulled out in the scoping review, it would seem this is a topic that has been widely researched in Kenya (>250 articles meeting criteria).

Line 58- Please write ‘PRISMA’ in full first time it is used

The scoping review covers until January 2022 which is more than 1 year ago. Would the authors want to update this search to be more current. This is especially so because it seems this is a widely researched topic.

Conclusions line 69- Could it be that the minimal research on MCL and PKDL in Kenya is because these forms are rare? In fact, this has been acknowledged by the authors later in the manuscript (line 136/316). Please explain

*Introduction*

Line 106- Non-specific presentation is mainly common in the early stages of the disease. The later stages are almost clinically pathognomonic e.g. the disfiguring skin lesions of CL/MCL and the organ enlargement of VL. The authors should consider making this clear. In the middle- and low-income countries, lack of diagnostics is a major hindrance to early treatment.

Line 117- It would be important for the authors to indicate that most of the drug treatments are expensive and unavailable in places like Kenya, where hospitals rely on donations from WHO for these medications. This is important for context.

Line 134- The authors should consider citing some of the primary literature on CL in Kenya e.g. <https://journals.plos.org/plosntds/article?id=10.1371/journal.pntd.0007712>, <https://pubmed.ncbi.nlm.nih.gov/31971945/>,

*Methods*

Line 157- Please proofing the sentence for correct grammar. The word ‘and’ could have been missed.

Article inclusion and exclusion criteria- The authors should specify if dissertations were included among the eligible articles. If not, the authors should justify

Line 191- Please explain what is meant by “Themes”. How were these established? Please provide more information on the basis of categorization of the articles by themes

*Results, discussion and implications of the findings to policy makers*

The authors should explicitly explain the basis of categorizing the articles in the various themes and sub groups, and why it is important for this paper. Ordinarily, some fields of research would attract fewer papers (e.g. reviews, since one article usually summarizes many other publications). Therefore the metrics of comparison using proportions would be misleading, since some of the fields would naturally be underrepresented.

The authors should explain if they also considered studies on modeling the burden/distribution, economic costs and risk mapping of Leishmaniasis in Kenya. Such studies would be important for the elimination efforts in Kenya. This scoping review is silent on any of these types of studies. This major limitation should be acknowledged by the authors.

This paper highlights the areas in Leishmaniasis research that are under-studied. It would be even better if the actual gaps in the research were highlighted. For example, it would be important to know what actual gaps are highlighted under diagnostics, treatment, prevention etc, as these will be more useful for the Leishmaniasis control effort in Kenya. The authors should update to include this information or explain why this data is not included.

Line 303- data from DHIS2 is aggregated, and is possible that same patient with CL lesions/VL symptoms could be reported each time they visit the hospital for follow up. Therefore, this dataset would not be accurate for highlighting the ‘apparent increased burden’ of Leishmaniasis

The authors should summarize the implications of their findings to policy and prevention efforts in Kenya

*Figures and tables*

Consider removing the % labels on the data in fig 4A and 4B

S3 table- please perform a grammar check on the table cells to remove errors. For example, the word ‘given’ is misspelt as ‘gjven’
